# The Root Hair Development of Pectin Polygalacturonase PGX2 Activation Tagging Line in Response to Phosphate Deficiency

**DOI:** 10.3389/fpls.2022.862171

**Published:** 2022-05-02

**Authors:** Qing Zhang, Aiwen Deng, Min Xiang, Qiuyan Lan, Xiaokun Li, Shuai Yuan, Xin Gou, Shuang Hao, Juan Du, Chaowen Xiao

**Affiliations:** Key Laboratory of Bio-Resource and Eco-Environment of Ministry of Education, College of Life Sciences, Sichuan University, Chengdu, China

**Keywords:** cell wall, pectin, polygalacturonase, phosphate deficiency, root hair development, *Arabidopsis thaliana*

## Abstract

Pectin, cellulose, and hemicellulose constitute the primary cell wall in eudicots and function in multiple developmental processes in plants. Root hairs are outgrowths of specialized epidermal cells that absorb water and nutrients from the soil. Cell wall architecture influences root hair development, but how cell wall remodeling might enable enhanced root hair formation in response to phosphate (P) deficiency remains relatively unclear. Here, we found that POLYGALACTURONASE INVOLVED IN EXPANSION 2 (PGX2) functions in conditional root hair development. Under low P conditions, a *PGX2* activation tagged line (*PGX2^AT^*) displays bubble-like root hairs and abnormal callose deposition and superoxide accumulation in roots. We found that the polar localization and trafficking of PIN2 are altered in *PGX2^AT^* roots in response to P deficiency. We also found that actin filaments were less compact but more stable in *PGX2^AT^* root hair cells and that actin filament skewness in *PGX2^AT^* root hairs was recovered by treatment with 1-N-naphthylphthalamic acid (NPA), an auxin transport inhibitor. These results demonstrate that activation tagging of *PGX2* affects cell wall remodeling, auxin signaling, and actin microfilament orientation, which may cooperatively regulate root hair development in response to P starvation.

## Introduction

Phosphate (P) is an essential mineral macronutrient for the normal growth and development of plants. In nature, phosphate widely exists in insoluble organic compounds in the soil, and soluble inorganic P is relatively unavailable for uptake by plants (Holford, 1997). Therefore, limited phosphate availability negatively affects global crop yields (Lopez-Arredondo et al., 2014). In P-deficient soil, plants can adapt to acquire this nutrient by reorganizing their root system architectures (Lopez-Bucio et al., 2002; Peret et al., 2014). These root architectural changes include the development and growth of primary roots, lateral roots, and root hairs, which allow plants to better adapt to soil environments (Bates and Lynch, 1995; Williamson et al., 2001; Lopez-Bucio et al., 2002). Root hairs expand out from root epidermal cells and facilitate the uptake of water and nutrients from the soil by increasing the root surface area. In P-deficient conditions, plants can increase root hair number and length to enhance overall root absorption capacity and efficiency (Bates and Lynch, 2000). Thus, root hairs play a crucial role in plant survival under nutrient starvation conditions.

The hairs that extend from root hair cells, or trichoblasts, exhibit polarized growth in which cell length is much greater than cell width, making root hairs a good model for investigating anisotropic cell expansion and polarity (Hepler et al., 2001). Root hairs initiate from bulges that form on the rootward side of the outer periclinal walls of trichoblasts, followed by the establishment of tip growth from these bulges. The formation of root hairs is collectively determined by the transcriptional regulation of cell fate, hormones, and polarity factors (Balcerowicz et al., 2015). Two classes of proteins Rop GTPase and respiratory burst oxidase homolog (RBOH) have been shown to function in root hair growth in Arabidopsis. Rop2 GTPase acts as a positive regulatory switch in root hair initiation (Jones et al., 2002). RbohC/RHD2 derives reactive oxygen species (ROS) activity to regulate root hair elongation in a Ca^2+^-dependent manner (Foreman et al., 2003; Takeda et al., 2008; Monshausen et al., 2009). An increasing number of research reveals that phytohormone auxin plays a key role in promoting root hair formation and elongation by triggering cell division and expansion (Lopez-Bucio et al., 2002; Kapulnik et al., 2011; Bhosale et al., 2018). Several auxin response mutants of *aux1*, *axr1*, *axr2,* and *axr3* in Arabidopsis have a pronounced effect on root hair length, indicating that auxin is required for normal root hair elongation (Pitts et al., 1998). Auxin can regulate *ROOT HAIR DEFECTIVE 6-LIKE 2* (*RSL2*) gene expression and ROS homeostasis to affect root hair growth (Mangano et al., 2018). Auxin can also induce receptor-like kinase ERULUS (ERU) activity to control root hair growth by modulating pectin dynamics (Schoenaers et al., 2018). Meanwhile, auxin integrates hormonal pathways and environmental factors to modulate root hair growth (Lee and Cho, 2013). Importantly, phosphate availability causes changes in hormone sensitivity in Arabidopsis root hair (Lopez-Bucio et al., 2002), and the activities of PIN-FORMED (PIN) proteins are involved in regulating root hair cell morphology (Ganguly et al., 2010). Mutants disrupting auxin synthesis (TRYPTOPHAN AMINOTRANSFERASE OF ARABIDOPSIS1, TAA) and transport (AUXIN RESISTANT1, AUX1) genes attenuate the response of root hairs to low P conditions (Bhosale et al., 2018). Additionally, hormone synthesis, transport, and sensitivity in roots are affected by P-deficient nutrient conditions, and thus, root architecture is reorganized in response to changes of phosphate availability (Lopez-Bucio et al., 2002).

In the auxin signaling pathway, polar auxin movement depends on auxin influx carrier proteins such as AUX1 and PINs, which mediate auxin transport across the plasma membrane (Friml, 2010). Arabidopsis AUX1 transports auxin from non-hair cells to developing hair cells to sustain root hair outgrowth (Jones et al., 2009), and rice OsAUX1 functions to mobilize auxin from the root apex to the differentiation zone to promote root hair elongation (Giri et al., 2018). PIN proteins are required for organ formation and plant development (Sabatini et al., 1999; Adamowski and Friml, 2015; Lee et al., 2020). PINs regulate cell division and expansion by controlling auxin distribution in plant roots (Blilou et al., 2005; Glanc et al., 2018). The expression of several PINs including PIN1, PIN2, and PIN3 in root hair cells greatly inhibits root hair growth, most likely by changing auxin level and PIN trafficking in the root hair cells (Ganguly et al., 2010). PIN2 generates an auxin gradient in epidermal cells that is crucial for root polarity (Nacry et al., 2005; Abas et al., 2006), and PIN3 is involved in both root hair growth and lateral root development (Lee and Cho, 2006; Chen et al., 2015). Auxin response factors (ARFs) control auxin-mediated transcriptional regulation of auxin-responsive genes (Tiwari et al., 2003), and ARF7 and ARF19 in Arabidopsis modulate root formation in response to P starvation (Huang et al., 2018). In addition to the role of auxin on root hair formation, actin cytoskeleton is also required for root hair morphogenesis (Kost et al., 1999; Hussey et al., 2006; Scheuring et al., 2016). The organization of actin microfilaments and their dynamics are essential for regulating cell expansion during root hair formation (Baluska et al., 2000; Wan et al., 2017; Wang et al., 2020); rapid cell elongation is concomitant with highly dynamic actin reorganization in roots (Takatsuka et al., 2018). Therefore, both auxin and actin drive root hair morphogenesis.

In plants, cell size and shape are determined by the deposition and mechanics of cell walls. The deposition of polysaccharides, including cellulose, hemicelluloses, and pectins, and structural remodeling of the wall are required for root cell formation (Park et al., 2011; Pena et al., 2012; Schoenaers et al., 2018). Some genes that control cell wall formation are involved in regulating root growth. Cellulose synthase-like proteins and xyloglucan-modifying proteins called xyloglucan endotransglucosylases/hydrolases (XTHs) have been shown to function in root growth (Favery et al., 2001; Vissenberg et al., 2005; Park et al., 2011). The modification and degradation of pectins mediated by pectin methylesterases (PMEs), pectin methylesterase inhibitors (PMEIs), and polygalacturonases (PGs) directly control the physicochemical properties of pectin, thereby influencing root growth (Levesque-Tremblay et al., 2015; Geng et al., 2017; Schoenaers et al., 2018).

Transcriptome data have shown that the expression of pectin-related genes changes in the roots of auxin-deficient Arabidopsis mutants or in response to low phosphate conditions (Yi et al., 2010; Hoehenwarter et al., 2016). Salazar-Haneo et al. used a coexpression-based approach and identified a class of P deficiency-induced genes encoding cell wall-modifying proteins that potentially function in root hair development (Salazar-Henao et al., 2016). Among these, a pectin degradation gene, *POLYGALACTURONASE INVOLVED IN EXPANSION 1* (*PGX1*), was predicted to respond to P starvation (Salazar-Henao et al., 2016); PGX1 was previously found to function in hypocotyl elongation and flower development in Arabidopsis (Xiao et al., 2014). Our previously studies showed that overexpression of *PGX2* in *PGX2^AT^* seedlings inhibits primary root growth (Xiao et al., 2017), and alteration of *PGX3* gene expression affects stomatal development and rosette expansion (Rui et al., 2017). In addition, *ARABIDOPSIS DEHISCENCE ZONE POLYGALACTURONASE1* (*ADPG1*) and *ADPG2* regulate floral organ abscission and anther dehiscence (Ogawa et al., 2009). Recently, another polygalacturonase, PG45 functions in leaf curvature (Yang et al., 2021). These characterized polygalacturonase genes are from different clades of phylogenetic tree (Xiao et al., 2014) and play important functions in multiple different organs during plant growth and development. However, the molecular mechanisms by which pectin degradation is associated with root growth in response to P deficiency remain elusive.

In an effort to understand how pectin degradation affects root hair development in response to P deficiency and auxin signaling, seedlings were grown under low P conditions or treated with 1-naphthaleneacetic acid (NAA) and 1-N- naphthylphthalamic acid (NPA), to observe the responses of root hairs to P availability. We found that the *PGX2* activation tagged line (*PGX2^AT^*) but not overexpression and mutant lines of other tested PG genes displayed a phenotype of bubble-like root hairs under low P conditions, which was consistent with results from pectinase treatments *in vitro*. The activation tagging of *PGX2* triggered the production of superoxide and inhibited callose and anthocyanin accumulation in *PGX2^AT^* seedlings. Both NAA and NPA application inhibited the formation of bubble-like root hairs in *PGX2^AT^* roots. The trafficking and distribution of the auxin efflux carrier PIN2 were also altered in the roots of *PGX2^AT^* seedlings but were rescued with P deficiency. Moreover, actin filaments were less bundled and more stable in *PGX2^AT^* root hair cells than in the Col controls. In addition, we also found that *PGX2* transcript was downregulated in auxin pathway mutants. Together, these data link cell wall remodeling, the phytohormone auxin, and the actin cytoskeleton in regulating root hair morphogenesis in response to P starvation.

## Materials and Methods

### Plant Materials and Growth Conditions

*Arabidopsis thaliana* Columbia (Col-0) ecotype was used as the wild-type control in this study. The *PGX2^AT^* activation tag line and *PGX1^OE-1^*, *PGX1^OE-48^*, *PGX3^OE^*, *pgx1-1* (WiscDsLox262B06), *pgx1-2* (Salk_026818), *pgx2* (Salk_071023), and *pgx3* (Salk _010192) plants were described on previously published papers (Xiao et al., 2014, 2017; Rui et al., 2017). Transgenic plants were constructed by transforming *Lifeact-GFP* into Col and *PGX2^AT^* plants with *Agrobacterium tumefacien*s strain GV3101 using the floral dipping method (Clough and Bent, 1998). Positive transformants were screened on half-strength (½) MS plates containing 2.2 g/l MS salts (Caisson Laboratories, Cat#M519), 0.6 g/l MES, 1% (w/v) sucrose, 0.8% (w/v) agar-agar, and 25 μg/ml hygromycin (Phyto Technology, Cat#H397). *PIN2-GFP PGX2^AT^* plants were generated by crossing *PGX2^AT^* with *PIN2-GFP* plants. Homozygous plants were identified by GFP fluorescence screening. Sterilized seeds were sown on ½ MS plates for 3 days; then, seedlings were transferred into normal ½ MS plates (+P, 480 μm) or low-phosphorus ½ MS (MS salt without phosphate, Caisson Laboratories, Cat#MSP11) plates supplemented with 10 μm KH_2_PO_4_ and 49.5 μm K_2_SO_4_ (-P, 10 μm) for another 4 days. Seedlings were grown in a 22°C chamber with a 16-h-light (100 to 150 μmol m^−2^ s^−1^)/8-h-dark lighting regime, and adult plants in soil were grown in a greenhouse under the same lighting conditions.

### Root Hair Growth Phenotype

To measure primary root length, Arabidopsis seedlings grown on ½ MS plates were scanned with an HP Scanjet G4050 scanner and primary root lengths were measured using ImageJ. For measurements of root hair length, seedlings were photographed directly using a stereomicroscope (Leica M205FA), and root hair lengths were measured using ImageJ. To clearly observe root hair phenotype, root hairs were imaged using an epiflorescence microscope (Leica DM4B), and the width of each root hair base was measured using ImageJ and the number of root hairs with bubble-like morphology was counted. To observe the growth phenotype of root hairs after NPA and NAA treatments, 3-day-old seedlings grown on ½ MS plates containing 5 μm NPA (Sigma-Aldrich, Cat#33371) or 50 nm NAA (Sigma-Aldrich, Cat#N0640) were transferred into normal or low-phosphorus ½ MS plates supplemented with 5 μm NPA or 50 nm NAA to grow for another 4 days. Roots hairs were photographed under an epiflorescence microscope (Leica DM4B), and GFP fluorescence from *Lifeact-GFP* seedlings was detected using a spinning-disk confocal microscope with a 488-nm excitation laser and 525/50-nm emission filter with a 100x objective (Zeiss, Observer SD).

### Gene Expression Analysis

Seedlings were collected and total RNA was extracted using a Plant RNA Kit (Omega). RNA concentration was measured by spectrophotometer (NanoDrop^™^ One^C^), and first-strand cDNA was synthesized using qScript cDNA SuperMix (Takara) with 500 ng DNase I-treated RNA. qPCR was performed using SYBR Green FastMix (Takara) with cDNA and gene-specific primers on a Bio-Rad CFX96 Touch Real-Time PCR machine. *ACT2* or *EF1α* was amplified as an internal control. Data were analyzed using Bio-Rad CFX manager software and relative gene expression levels were calculated relative to *ACT2* or *EF1α* using the ΔΔCT method. Genes and primer sequences used for qPCR are listed in Supplementary Tables 1, 2.

### Chemical Treatments

For exogenous pectinase treatment, Arabidopsis seedlings grown for 4 days after transferring on normal or P-deficient ½ MS medium plates were collected, and 7-day-old seedlings were incubated with liquid ½ MS medium containing 25 U/ml pectinase (Sigma-Aldrich, Cat#P2611) for 20 min at 30°C. Then, seedlings were washed by liquid ½ MS medium and photographed under an epifluorescence microscope (Leica DM4B).

To detect callose deposition in roots, aniline blue staining was performed according to a previously described method (Muller et al., 2015). Briefly, 7-day-old seedlings was stained with 0.1% (w/v) aniline blue (Sigma-Aldrich, Cat#415049) in 100 mm phosphate buffer (pH 7.2) for 1.5 h. Root samples were placed in 20% (v/v) glycerol to observe under a spinning-disk confocal microscope with a 405-nm excitation laser and 445/50-nm emission filter with a 20x objective (Zeiss, Observer SD).

### PI Staining

To clearly observe root hair phenotype, 7-day-old Col and *PGX2^AT^* seedlings grown under normal and phosphate deficient conditions were stained with 1 ml 20 μg/ml propidium iodide (PI, Life Technologies, Cat#P3566) solution for 20 s in dark. The images of stained root hairs were recorded using a spinning-disk confocal microscope with a 561-nm excitation laser and 605/70-nm emission filter with a 100x objective (Zeiss, Observer SD). For superoxide detection, 7-day-old seedlings were incubated in 100 mm phosphate buffer (pH 7.2) containing with 0.5 mg/ml Nitro Blue Tetrazolium (NBT; Sigma-Aldrich, Cat#N6876) for 45 min, and images were recorded using a stereomicroscope (Leica M205FA).

Brefeldin A (BFA) treatment was performed as previously described method (Lin et al., 2020) with minor modifications. Seven-day-old seedlings grown on ½ MS medium plates were transferred to liquid ½ MS medium containing 50 μm BFA (Abmole, Cat#M2294) and incubated for 30 min. Seedlings were then washed with liquid ½ MS medium for 1.5 h. The roots were observed and imaged under a spinning-disk confocal microscope (Zeiss, Observer SD). Cells with visible BFA bodies were counted from epidermal cells of seedling roots. For actin inhibitor treatment, seedlings were incubated in liquid ½ MS medium containing 100 nm Latrunculin A (Lat A, Thermo Fisher, Cat#L12370) for 1 h, then washed by liquid ½ MS medium, and images of actin filaments were taken using a spinning-disk confocal microscope with a 488-nm excitation laser and 525/50-nm emission filter with a 100x objective (Zeiss, Observer SD).

### Immunolabeling

Seven-day-old Arabidopsis Col and *PGX2^AT^* seedlings grown on normal or P-deficient ½ MS medium plates were soaked in an isotonic fixation buffer containing 50 mm PIPES, 5 mm MgSO_4_, 5 mm EGTA, 4% (v/v) paraformaldehyde, 0.1% (v/v) glutaraldehyde, and 1% (w/v) sucrose, pH 6.9 for 30 min, then transferred to the same fixation buffer for 90 min, and subsequently washed with 50 mm PIPES buffer (pH 6.9) three times. Seedling samples were blocked with PIPES buffer containing 3% (w/v) non-fat milk for 1 h. After washing by PIPES buffer three times, seedlings were incubated with a primary antibody (Agrisera; JIM5, Cat#AS184194; JIM7, Cat#AS184195; 1:10) at 4°C overnight. After washing by PIPES buffer, the samples were incubated with Alexa Fluor 488-conjugated goat anti-rat IgG secondary antibody (KPL, Cat#5230; 1:500) for 1 h at room temperature. The samples were then mounted on a slide in PIPES buffer and visualized using a spinning-disk confocal microscope with a 488-nm excitation laser and 525/50-nm emission filter with a 100x objective (Zeiss, Observer SD). The quantification of fluorescence intensity in immunolabeling images was performed by ZEN software (Zeiss), and the selected regions were used to quantify fluorescence intensity and the final fluorescence value was subtracted the background value.

### Measurements of Uronic Acids and Methanol Release

Preparation of alcohol insoluble residue (AIR) enriched in cell walls and measurements of uronic acid content and pectin methylesterification degree were performed as described by Du et al. (2020).

### Confocal Imaging of Lifeact-GFP and PIN2-GFP

Images of Lifeact-GFP and PIN2-GFP in root hairs and root tips were recorded under a spinning-disk confocal microscope with a 488-nm excitation laser and 525/50-nm emission filter with a 100x objective (Zeiss, Observer SD). Parameters representing actin filament density, skewness, and orientation were analyzed and quantified by ImageJ (Higaki, 2017). Briefly, original images were opened, cell regions were manually segmented, and cell region area and the angle of actin filaments relative to the cell growth axis were measured. Skeletonized images were obtained from the original images and masked with manually segmented cell region images. The parameters of actin filaments were quantified using the masked skeleton images. Fluorescence signal intensity of PIN2-GFP at the plasma membrane (PM) and intracellular compartments was quantified by ImageJ (Jensen, 2013). PM-associated GFP fluorescence was determined in regions of interest (ROIs) using the rectangle and/or freehand line tools in ImageJ. The intracellular signal of PIN2-GFP was determined by measuring multiple ROIs as the total intracellular fluorescence intensity, which excludes signals from the nuclei and nonspecific background. The resulting data were normalized to the mock control, and the signal ratio of PM to intracellular compartments was presented. For quantification of the BFA-induced internalization of PIN2-GFP, the level of internalized PIN2-GFP was presented as the number of GFP-labeled BFA bodies per cell (French et al., 2008).

## Results

### A *PGX2^AT^* Activation Tagged Line Has Bubble-Like Root Hairs Under P-Deficient Conditions

**Figure 1:**
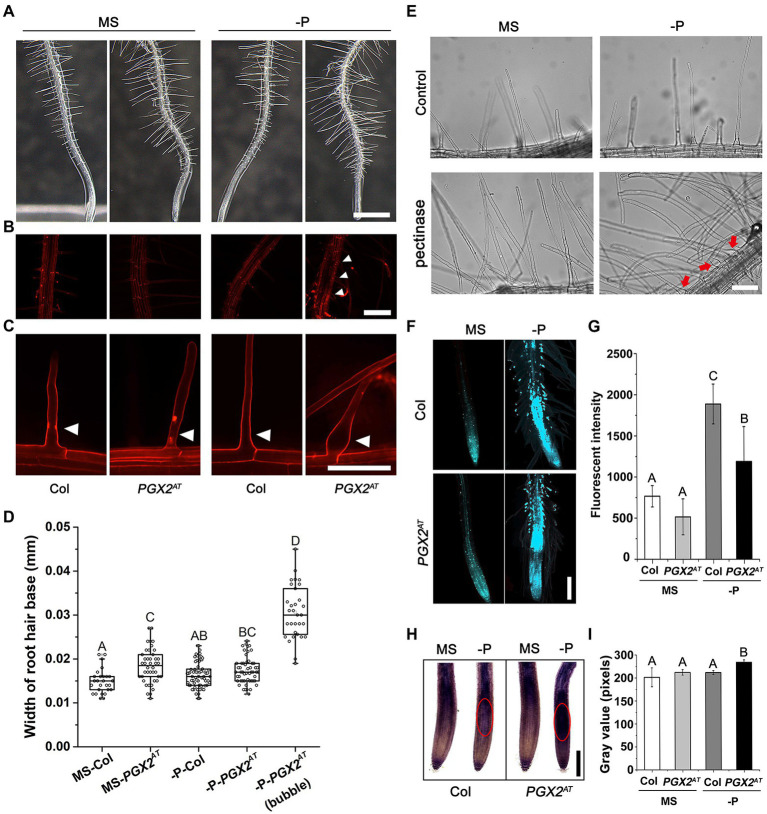
Root hairs of *PGX2* activation tag (*PGX2^AT^*) seedlings are sensitive to low phosphate conditions. **(A)** Primary roots with root hairs of 7-day-old Col and *PGX2^AT^* seedlings grown in ½ MS medium plates under normal (MS) and phosphate deficient (-P) conditions. Three-day-old seedlings were transferred from normal MS plates into normal MS or -P conditions for another 4 days. Images were taken on the 4th day after seedling transfer. Bar = 1 mm. **(B)** Zoomed in images from **(A)** focusing on root hair regions by PI staining. Bar = 200 μm. **(C)** PI staining of root hairs in Col and *PGX2^AT^* seedlings. Red and white arrowheads indicate positions of root hair bases. Bar = 0.1 mm. **(D)** Widths of root hair bases in Col and *PGX2^AT^* seedlings grown in normal MS or -P conditions (*n* ≥ 27 root hairs). **(E)** Pectinase treatment of Col seedlings after transferring to normal MS or -P conditions to grow for 4 days. Red arrows indicate bubble-like root hair bases. Bar = 100 μm. **(F)** Aniline blue staining for callose in roots of Col and *PGX2^AT^* seedlings. Images were taken on the 4th day after seedling transfer. Bar = 200 μm. **(G)** Arbitrary fluorescent intensity of images for aniline blue staining (*n* = 10 seedlings per each genotype). **(H)** NBT staining for superoxide in root tips of Col and *PGX2^AT^* seedlings. Red circles show highly stained regions. Images were taken in the 4th day after seedling transfer. Bar = 200 μm. **(I)** Gray value of NBT color intensity (*n* = 10 seedlings per each genotype). Error bars represent SD. Uppercase letters indicate significantly different groups as determined by one-way ANOVA with *post-hoc* Duncan’s test (*p* < 0.05).

Plants can respond quickly to phosphate deficiency by increasing root hair length and density to enhance phosphate acquisition (Lopez-Bucio et al., 2002; Lynch, 2011). Cell wall remodeling plays a critical role in this process (Ogden et al., 2018). To explore how cell wall remodeling mediated by pectin-degrading polygalacturonases (PGs) affects root hair development, we screened a series of PG-related mutants for root hair phenotypes in response to P deficiency. Firstly, we detected gene expression in roots of *PGX1^OE1^*, *PGX3^OE^*, and *PGX2^AT^* seedlings. qPCR results showed that *PGX1* expression was comparable to wild type, *PGX3* expression was enhanced in *PGX3^OE^* line, and *PGX2* was slightly increased in the roots of *PGX2^AT^*, but its expression was significantly enhanced in the whole *PGX2^AT^* seedlings (Supplementary Figures 1A–D). Neither overexpression nor loss of function for two polygalacturonase genes, *PGX1* and *PGX3*, showed significantly different root hair phenotypes compared with wild-type Col controls (Supplementary Figure 1E). The *PGX2* activation tagged line (*PGX2^AT^*) displayed obvious bubble-like root hairs in response to P deficiency (Figures 1A–C), whereas the morphology of root hairs in *pgx2* mutant was similar to that of Col control (Supplementary Figure 1E). *PGX2^AT^* seedlings had longer root hairs under normal and phosphate deficient conditions with similar growth rate (Supplementary Figure 2). We next quantified the width of the root hair base and found that *PGX2^AT^* root hairs were wider than Col under normal conditions, and root hairs were the widest in P-deficient conditions (Figure 1D). To confirm that the bubble-like phenotype of root hairs resulted from pectin degradation in response to P deficiency, we incubated Col seedlings in liquid MS media with exogenous pectinase. The results revealed that the application of exogenous pectinase facilitated the formation of irreversibly bubble-like root hairs in Col seedlings grown under P-deficient conditions (Figure 1E). We also observed that the primary roots of *PGX2^AT^* seedlings were shorter than those of Col in both normal and P-deficient conditions (Supplementary Figures 3A,B), whereas P deficiency has a similar negative effect on root growth in both genotypes (Supplementary Figure 3C). These data reveal the activation tagging of *PGX2* promotes cell expansion in root hairs.

In order to further investigate the response of *PGX2^AT^* seedlings to P deficiency, we performed aniline blue and NBT staining in roots and found that callose deposition was decreased in root hairs, and superoxide accumulated more in root tips of *PGX2^AT^* seedlings than in Col controls (Figures 1F–I). In general, P deficiency triggers anthocyanin accumulation in wild-type Arabidopsis (Jiang et al., 2007); however, there was less anthocyanin accumulation in *PGX2^AT^* seedlings than in Col controls under low P conditions (Supplementary Figure 4). These experimental results show that *PGX2^AT^* seedlings have different callose and anthocyanin deposition in response to nutrient stress.

### The Pectin Physiological and Biochemical Properties Are Altered in *PGX2^AT^* Line

**Figure 2:**
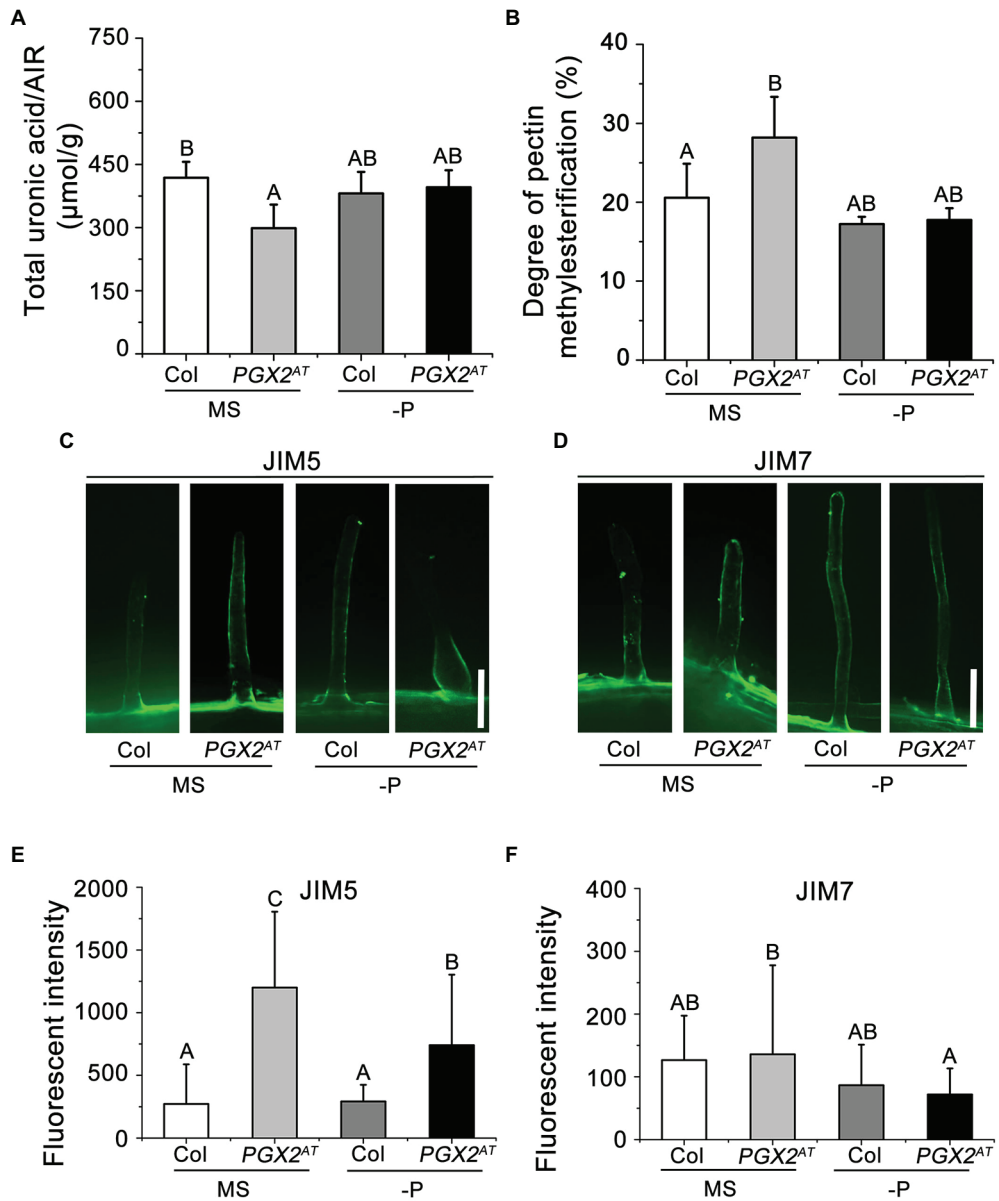
The pectin content and degree of pectin methylesterification are altered in *PGX2^AT^* seedlings. **(A,B)** Total uronic acid content and degree of pectin methylesterification of seedlings grown in normal MS and low phosphate (-P) conditions (*n* = 5 technical replicates). **(C,D)** Immunolabeling in root hair cells of Col and *PGX2^AT^* seedlings after transferring for 4 days by JIM5 and JIM7, respectively. Bars = 50 μm. **(E,F)** Arbitrary fluorescent intensity of immunolabeling images for JIM5 and JIM7 antibodies (*n* ≥ 13 root hairs per each genotype). Error bars represent SD. Uppercase letters on top of bar charts indicate significantly different groups as determined by one-way ANOVA with *post-hoc* Duncan’s test (*p* < 0.05).

In our previous studies, PGX1, PGX2, and PGX3 have been characterized as polygalacturonases that cleave pectin molecules and modulate demethylesterified HG abundance (Xiao et al., 2014, 2017; Rui et al., 2017). To determine whether PGX2-mediated pectin degradation affects pectin accumulation and pectin methylesterification, we measured uronic acid content and methanol released from the cell wall, which are used to calculate the degree of pectin methylesterification. Total uronic acid content in the cell walls of *PGX2^AT^* seedlings was significantly lower, while the degree of pectin methylesterification in *PGX2^AT^* seedlings increased compared to Col controls (Figures 2A,B). However, these differences were attenuated when seedlings were grown under P-deficient conditions (Figures 2A,B). To further examine whether the pectin modification is affected in the root hair wall of *PGX2^AT^* seedlings, we performed immunolabeling experiments with JIM5 and JIM7 antibodies, which recognize low- and high-methylesterified HG, respectively (Willats et al., 2001; Guillemin et al., 2005). There was higher JIM5 signal intensity in *PGX2^AT^* root hairs than in Col when seedlings were grown in both normal MS- and P-deficient conditions, whereas there was no obvious difference in JIM7 signal intensity (Figures 2C–F). These data suggest that PGX2 influences pectin degradation and modification, and that P deficiency might interfere in its effects to some degree in *PGX2^AT^* seedlings.

### Auxin Is Required to Maintain Normal Root Hair Width

**Figure 3:**
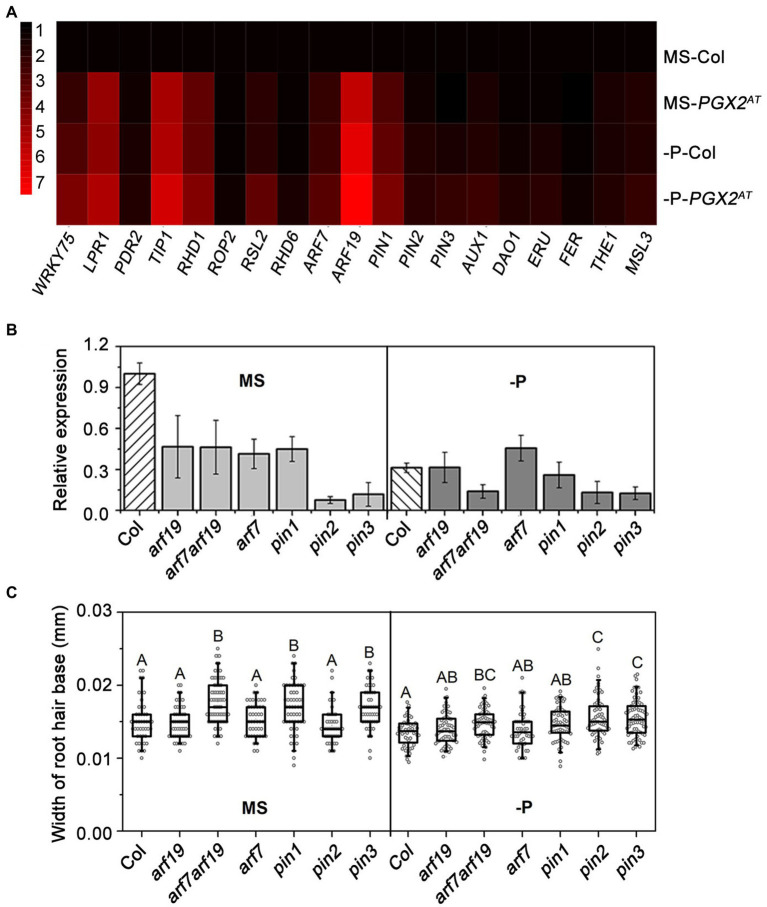
Relative gene expression level and width of root hair base. **(A)** Relative expression of genes related to phosphate deficient response, root hair development, auxin and cell wall integrity signaling by RT-qPCR in Col, and *PGX2^AT^* seedlings (*n* = 3 technical replicates). **(B)** Relative gene expression level of *PGX2* in auxin-related mutants with phosphate deficient (-P) conditions compared to MS controls (*n* = 3 technical replicates). **(C)** Widths of root hair bases of auxin-related mutants grown in MS control and phosphate deficient (-P) conditions (*n* ≥ 48 root hairs). All data were collected in seedlings 4 days after transfer. Uppercase letters on top of boxplots indicate significantly different groups as determined by one-way ANOVA with *post-hoc* Duncan’s test (*p* < 0.01).

Previous studies have shown that auxin plays an important role in root hair development by regulating cell expansion or cell division (Kapulnik et al., 2011; Bhosale et al., 2018; Schoenaers et al., 2018). Auxin promotes root hair growth in response to P deficiency in Arabidopsis and rice (Balzergue et al., 2017; Giri et al., 2018). Moreover, cell wall integrity signaling trigged by cell wall remodeling is closely related to hormone signaling pathway (Novakovic et al., 2018). PGX2 is a polygalacturonase to control cell expansion by pectin degradation (Xiao et al., 2017) and the *PGX2^AT^* activation tagged line displayed a bubble-like root hair phenotype when seedlings were grown under P-deficient conditions (Figure 1). To explore whether auxin is also involved in this process along with cell wall regulators, especially in response to P deficiency, we analyzed the expression of genes associated with auxin signaling, P deficiency response, root hair development, and cell wall integrity (Supplementary Table 2). Gene expression was measured by qPCR, which showed that the expression levels of *LOW PHOSPHATE ROOT 1* (*LPR1*), *TIP GROWTH DEFECTIVE 1* (*TIP1*), *ROOT HAIR DEFECTIVE 1* (*RHD1*), *PIN1*, *ARF7*, and *ARF19* were upregulated in *PGX2^AT^* seedlings grown under both normal and P-deficient conditions (Figure 3A). Importantly, *ARF19* showed the highest expression level of the genes measured in *PGX2^AT^* seedlings under low P conditions (Figure 3A). *ARF19* functions in the auxin-mediated transcription that controls root hair growth (Michniewicz et al., 2007; Schoenaers et al., 2018). The auxin transporter PIN proteins are central components of the auxin efflux machinery that precisely regulate polar auxin transport (Sabatini et al., 1999). In addition, several genes associated with root hair formation, such as *TIP1*, *RHD1*, and *ROOT HAIR DEFECTIVE 6-LIKE 2* (*RSL2*), were upregulated in *PGX2^AT^*, which was further enhanced with P deficiency (Figure 3A). However, there were no significant differences in the expression of *FERONIA* (*FER*), *THESEUS 1* (*THE1*), or *MECHANOSENSITIVE CHANNEL OF SMALL CONDUCTANCE LIKE 3* (*MSL3*) genes associated with cell wall integrity in Col seedlings under low P or in *PGX2^AT^* seedlings under normal or low P conditions (Figure 3A).

To next investigate whether changes in auxin signaling and auxin transport conversely affect *PGX2* gene expression, we evaluated *PGX2* transcript levels in auxin signaling- or transport-deficient *arf7*, *arf19*, *arf7arf19*, *pin1*, *pin2*, and *pin3* mutants by qPCR. Expression of *PGX2* in all mutants was reduced, especially in *pin2* and *pin3* mutants, when seedlings were grown on normal MS medium plates (Figure 3B). Under P-deficient conditions, *PGX2* gene expression was significantly decreased in *arf7arf19* mutants but was still lower in *pin2* and *pin3* mutants than in Col (Figure 3B). These results indicate that *PGX2* expression might be regulated by auxin. The base of the root hairs in these mutants was measured and *arf7arf19*, *pin1*, and *pin3* mutants had wider root hairs in normal MS medium, and *arf7arf19*, *pin2*, and *pin3* mutants had wider root hairs under P-deficient conditions than the Col controls (Figure 3C). Both *arf7arf19* and *pin3* mutants displayed increased root hair cell width in both normal and P-deficient conditions compared with Col, whereas *pin2* mutants had wider root hairs only under P deficiency conditions. These experimental results imply that the alteration of *PGX2* gene expression in *PGX2^AT^* seedlings may have an effect on auxin signaling and that *PGX2* expression may also be regulated by auxin during root hair formation.

### NAA and NPA Application Alleviates the *PGX2^AT^* Root Hair Phenotype Under P-Deficient Conditions

**Figure 4:**
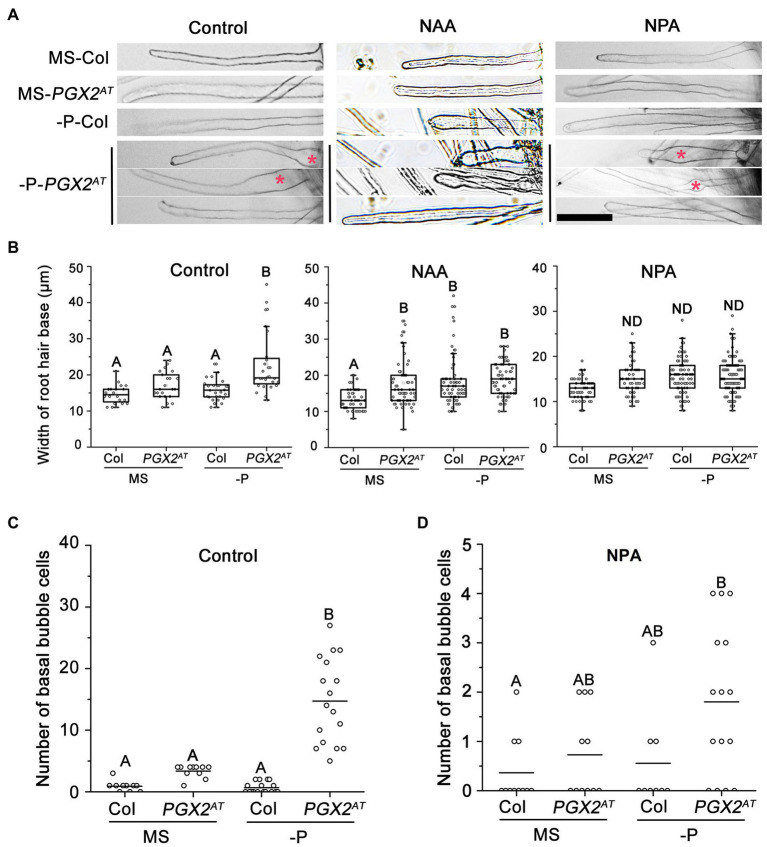
NAA/NPA treatments inhibit bubble-like phenotype in root hair bases of *PGX2^AT^* seedlings. **(A)** Both NAA and NPA treatments resulted in the occurrence of a few bubble-like root hairs at the top or middle under phosphate deficient (-P) conditions. Bars = 100 μm. **(B)** Widths of root hair bases in Col and *PGX2^AT^* seedlings grown in MS control and phosphate deficient (-P) conditions with NAA or NPA application (*n* ≥ 20 root hairs from at least 7 seedlings). **(C,D)** Number of basal bubble-like cells in Col and *PGX2^AT^* seedlings grown in MS control and phosphate deficient (-P) conditions without (Control) or with NPA application (*n* ≥ 9 seedlings). Uppercase letters indicate significantly different groups as determined by one-way ANOVA with *post-hoc* Duncan’s test (*p* < 0.01). ND, no statistical difference.

Our experimental results showed that *PGX2^AT^* seedlings have bubble-like root hair cells under P-deficient conditions (Figure 1). To assess the effect of auxin on the regulation of root hair shape, NAA or NPA was added into the growth medium. The application of 50 nm NAA or 5 μm NPA inhibited the bubble-like root hair phenotype of *PGX2^AT^* seedlings under P-deficient conditions; however, we occasionally observed bulging at the middle or tip of some root hairs under low P conditions in NPA-treated *PGX2^AT^* seedlings (Figure 4A). Quantification of basal bubble-like root hairs revealed that *PGX2^AT^* root hairs were wider after NAA treatment under normal growth conditions compared to control plants, but this difference was offset under low P treatment conditions (Figure 4B). Moreover, there was no significant difference in basal root hair width between Col and *PGX2^AT^* seedlings after NPA treatment under either normal or P-deficient conditions (Figure 4B) and NPA treatment markedly reduced the formation of bubble-like root hair cells under P-deficient conditions (Figures 4C,D).

We also measured root hair length of seedlings with NPA treatment under low P conditions. *PGX2^AT^* seedlings had longer root hairs than Col controls in both normal and P-deficient conditions, whereas NPA application facilitated longitudinal cell expansion in *PGX2^AT^* seedlings in response to P deficiency (Supplementary Figure 5). These results show that an imbalance in auxin levels induced the formation of bubble-like root hairs in *PGX2^AT^* plants, further highlighting the role of auxin and cell wall remodeling during root hair morphogenesis, especially in response to P deficiency.

### PIN2 Trafficking Is Altered in *PGX2^AT^* Seedlings in Response to P Deficiency

**Figure 5:**
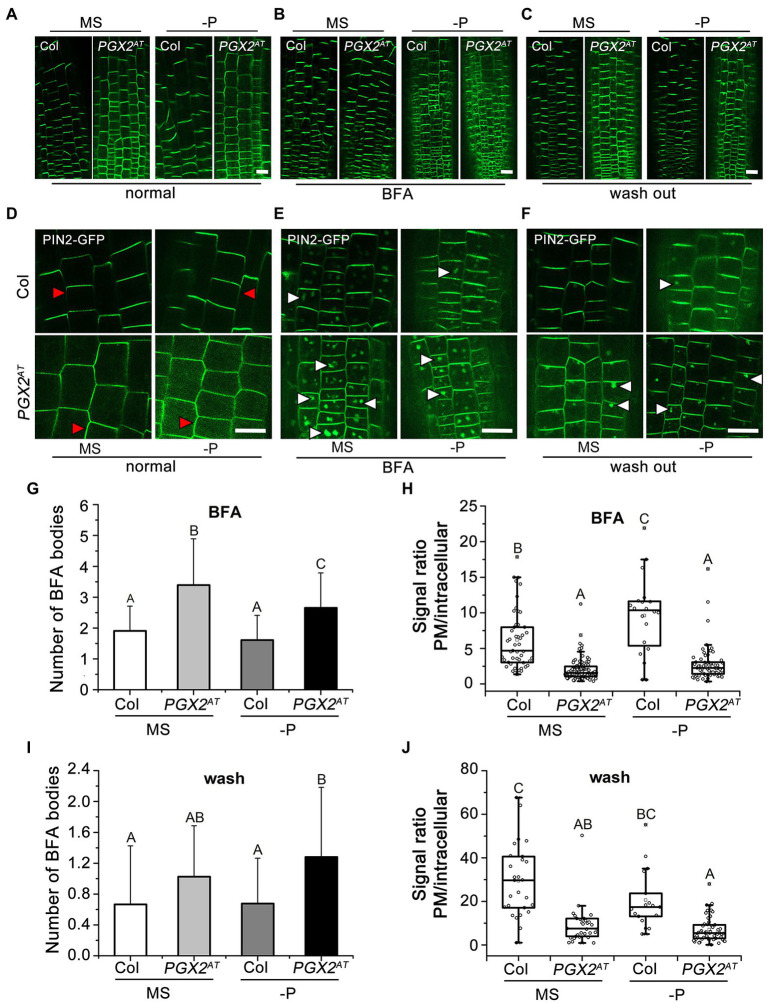
The abundance of plasma membrane (PM)-localized PIN2 protein is reduced in *PGX2^AT^* seedlings. **(A–C)** PIN2-GFP patterns in transgenic plants of *PIN2-GFP* Col and *PIN2-GFP PGX2^AT^* seedling roots. PIN2-GFP images were taken after transferring for 4 days in those seedlings before **(A)** and after **(B)** BFA treatment for 30 min, and after BFA was washed out for 90 min **(C)**. Bars = 50 μm. **(D–F)** Images recorded with higher magnification under a confocal microscope. Red arrowheads indicate plasma membrane-localized PIN2-GFP; white arrowheads indicate intercellular PIN2-GFP. Bars = 50 μm. **(G,H)** Numbers of BFA bodies and signal ratio of PM to intracellular PIN2-GFP with BFA treatments (*n* ≥ 50 cells from at least 10 seedlings). **(I,J)** Numbers of BFA bodies and signal ratio of PM to intracellular PIN2-GFP after washing BFA (*n* ≥ 28 cells from at least 10 seedlings). Uppercase letters indicate significantly different groups as determined by one-way ANOVA with *post-hoc* Duncan’s test (*p* < 0.01).

We showed that NAA and NPA treatments affected root hair morphology in *PGX2^AT^* seedlings (Figure 4) and that *PGX2* expression levels in *pin1*, *pin2*, and *pin3* mutants were decreased under both normal and P-deficient conditions (Figure 3B). These three mutants had wider root hairs, and the widths of root hair bases in *pin2* mutants significantly increased in response to P deficiency (Figure 3C). PIN2 functions in root hair development concomitantly with cellular trafficking and polar transport (Lee and Cho, 2013; Pandya-Kumar et al., 2014). This evidence prompted us to further investigate whether PIN2 localization and transport are disrupted in root cells of *PGX2^AT^* seedlings. We generated *35S::PIN2-GFP PGX2^AT^* plants by crossing *35S::PIN2-GFP* line to *PGX2^AT^*. We grew *35S::PIN2-GFP* Col and *35S::PIN2-GFP PGX2^AT^* seedlings on normal and P-deficient medium plates, and then analyzed GFP localization and intensity. We found that in *PGX2^AT^* seedling roots, PIN2-GFP was no longer polarized in root epidermal cells, especially in response to P deficiency, when compared with the polarized localization of PIN2-GFP in Col root cells (Figures 5A,D).

For auxin transport, the localization on plasma membrane (PM) of PIN proteins largely depends on dynamic vesicle recycling between the PM and endosomes (Dhonukshe et al., 2007). However, it is unknown whether the altered PIN2-GFP polarization in *PGX2^AT^* root cells is associated with changes in PIN2 recycling. Brefeldin A (BFA) is a vesicle-trafficking inhibitor that interferes with PIN recycling and induces the accumulation of PIN proteins in “BFA bodies” (Geldner et al., 2001). We incubated *35S::PIN2-GFP* Col and *35S::PIN2-GFP PGX2^AT^* seedlings with BFA for 30 min and then washed out the BFA for 1.5 h to allow PIN2 to repolarize in the plasma membrane (Figures 5B,C,E,F). After the application of BFA, there was a similar trend of increased BFA body number and reduced PM-localized PIN2-GFP fluorescence in *PGX2^AT^* root cells under both normal MS and P-deficient conditions (Figures 5G,H). The signal ratio of PM-localized to intracellular PIN2-GFP in *PGX2^AT^* root cells was lower than that in Col in response to P deficiency (Figure 5H). After BFA was washed out, the number of BFA bodies in *PGX2^AT^* root cells remained higher than in Col controls under P-deficient conditions (Figure 5I), and the fluorescent signal ratio of PM-localized to intracellular PIN2-GFP was lower under both normal and P-deficient conditions (Figure 5J). These results indicate that activation tagging of *PGX2* affects polar transport and distribution of PIN2 in the PM, and that this effect is enhanced by P deficiency in *PGX2^AT^* seedlings.

### Actin Filaments Are More Stable and Less Bundled in *PGX2^AT^* Than in Col Root Cells

**Figure 6:**
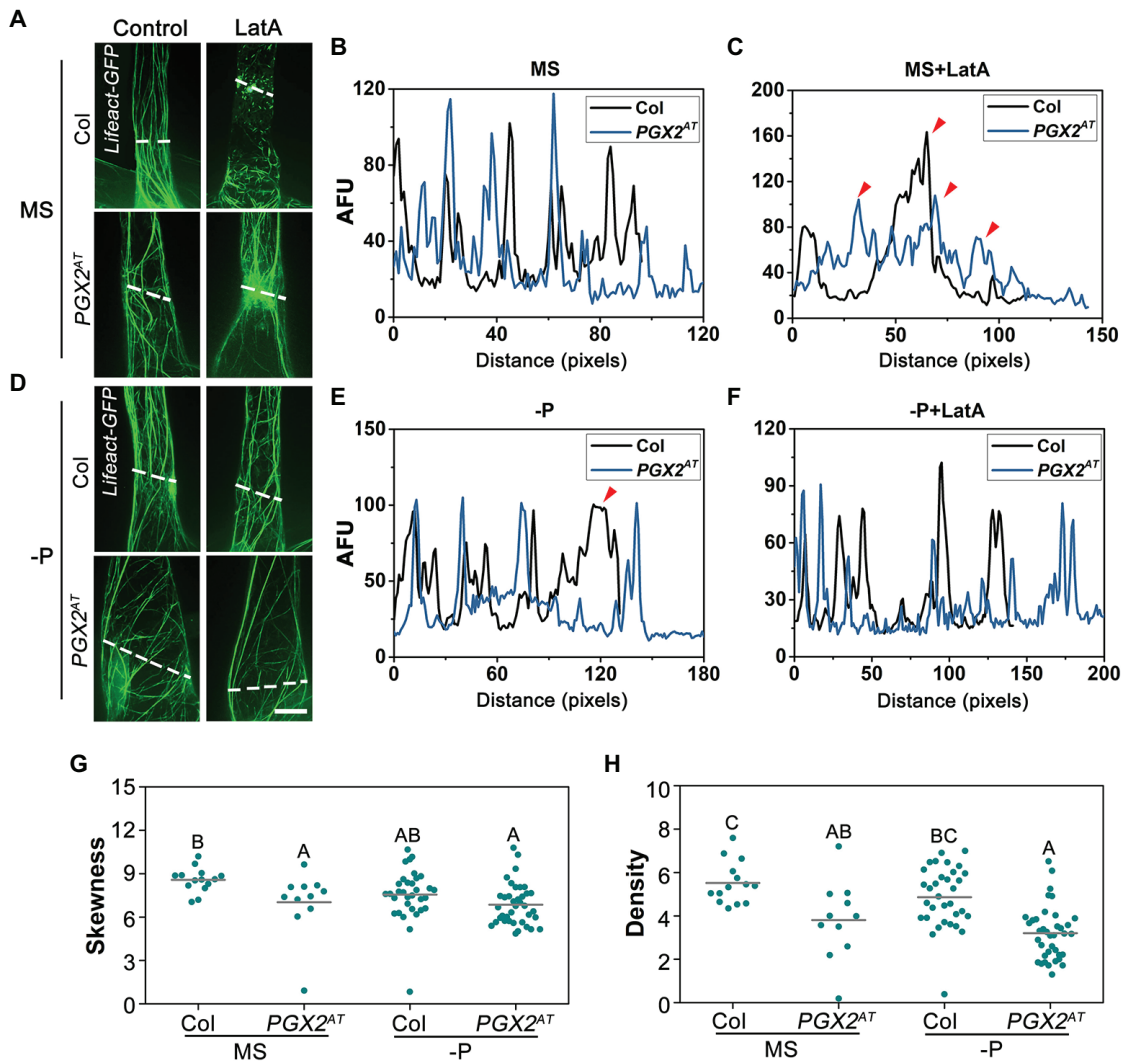
Actin filaments in root hair cells of *PGX2^AT^* seedlings show less skewness and more stable to LatA treatment. **(A,D)** *Lifeact-GFP* images of root hairs in 7-day-old *Lifeact-GFP* Col and *Lifeact-GFP PGX2^AT^* seedlings under normal and P-deficient conditions without (Control) or with 100 nm LatA treatment. Bar = 10 μm. **(B,C,E,F)** Profiles of fluorescent intensity along lines (perpendicular to cell growth axis) for Col (black line) and *PGX2^AT^* (blue line) cells in **(A)**. AFU, arbitrary fluorescence units. Arrows indicate differences between two lines. **(G,H)** Skewness and density of actin filaments decrease in *PGX2^AT^* seedlings (*n* ≥ 11 cells). Uppercase letters indicate significantly different groups as determined by one-way ANOVA with *post-hoc* Duncan test (*p* < 0.01).

The actin cytoskeleton is critical for vesicle trafficking and exocytosis in targeting PIN proteins to the plasma membrane during root hair development (Geldner et al., 2001; Ketelaar et al., 2003; Nagawa et al., 2012). Moreover, the organization and dynamics of actin filaments can influence root hair development in a PIN-independent fashion (Baluska et al., 2000; Pei et al., 2012; Ketelaar, 2013; Wang et al., 2020). Given the roles of the actin cytoskeleton and PGX2 in cell expansion and/or cell elongation (Xiao et al., 2017; Takatsuka et al., 2018; Arieti and Staiger, 2020), we generated transgenic plants expressing *Lifeact-GFP* in Col and *PGX2^AT^* backgrounds to examine the dynamics and sensitivity of actin filaments to LatA, which depolymerizes actin filaments. On normal MS medium, a few actin filaments in *PGX2^AT^* root hair cells were relatively intact after LatA treatment, whereas most actin filaments were fragmented in Col controls (Figures 6A–C). On P-deficient MS plates, actin filaments in both Col and *PGX2^AT^* root hair cells were more resistant to LatA treatment (Figures 6D–F). We also noticed that actin filaments in root hairs of *PGX2^AT^* seedlings grown on normal MS plates were more disorganized (Figure 6A) and that their distribution was different from that in Col root hairs under P-deficient conditions, which was possibly due to the bubble-like cell deformation of *PGX2^AT^* root hairs (Figure 6D). Next, we quantified the skewness, which represents actin filament bundling or aggregation and density, and is indicated by the dispersion of the Lifeact-GFP signal. The degree of skewness and density of actin filaments was lower in *PGX2^AT^* root hair cells than in Col controls (Figures 6G,H), indicating that actin filaments in *PGX2^AT^* root hairs are less bundled. However, the parameters of parallelness and angle that represent the direction of actin filaments did not show any difference between Col and *PGX2^AT^* root hairs (Supplementary Figures 6A,B). Together, these data suggest that PGX2-mediated cell wall remodeling can feed back on actin orientation and dynamics, which is affected by P-deficient stress.

### Low Actin Filament Skewness Is Rescued by NPA Application in *PGX2^AT^* Root Hairs

**Figure 7:**
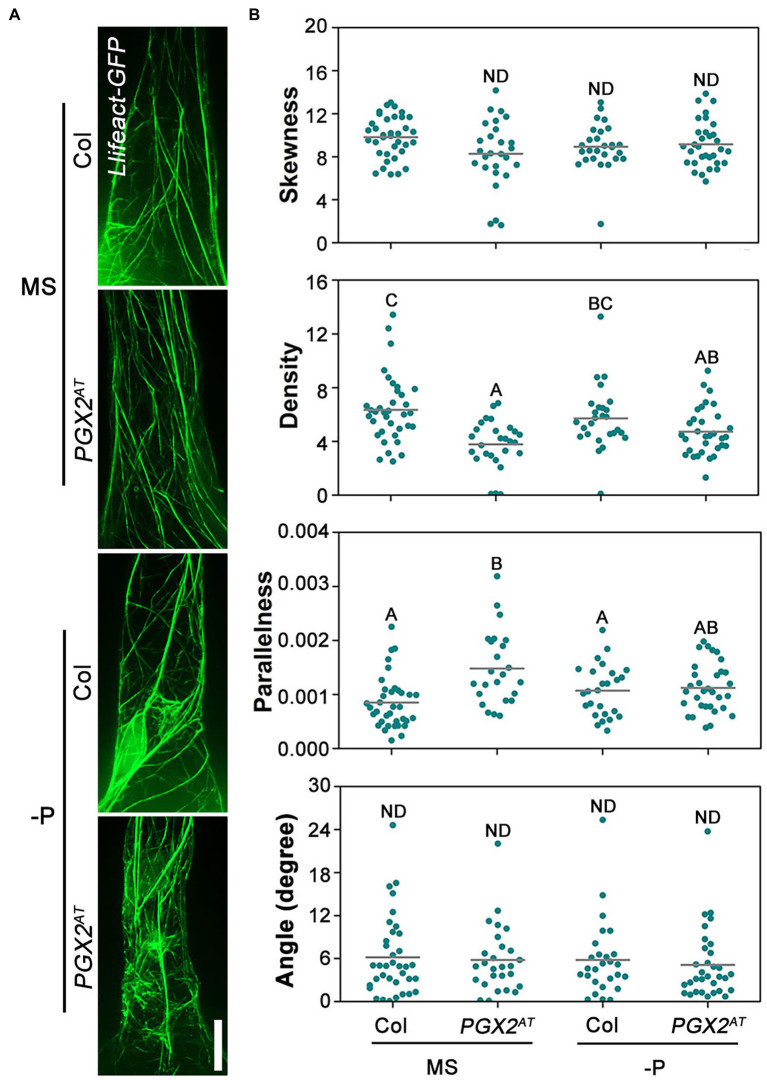
Actin filament skewness is recovered in *PGX2^AT^* root hair cells after NPA treatment. **(A)** Actin filaments in root hairs of *Lifeact-GFP* Col and *Lifeact-GFP PGX2^AT^* transgenic seedlings in the 4th day after transferring into normal MS or P-deficient conditions. The 5 μm NPA was added into media for seedling growth. Bars = 10 μm. **(B)** Skewness, density, parallelness, and angle of actin filaments were quantified by ImageJ (*n* ≥ 27 cells). Uppercase letters indicate significantly different groups as determined by one-way ANOVA with *post-hoc* Duncan testing (*p* < 0.01). ND, no statistical difference.

Auxin and auxin transport inhibitors have been shown to affect actin dynamics and cell growth in roots. Although NAA application can drastically inhibit cell elongation concomitantly with actin filament bundling, NPA treatment can reduce cell elongation, leading to partial depolymerization of actin filaments with punctuated structures (Michniewicz et al., 2007). To observe whether actin filaments labeled with *Lifeact-GFP* are affected by NPA treatment, we grew transgenic lines expressing *Lifeact-GFP* in the Col and *PGX2^AT^* genetic backgrounds on MS plates supplemented with 5 μm NPA for 7 days before imaging root cells by confocal microscope. The results showed a lower density and more parallel actin filaments in *PGX2^AT^* than in Col root hair cells, whereas there was no significant difference in skewness and angle of actin filaments between the two lines (Figure 7). It is worth noting that the decreased actin filament skewness in *PGX2^AT^* root hair cells was recovered with NPA application (Figure 7). Additionally, we did not observe bubble-like root hairs in *PGX2^AT^* seedlings after NPA treatment under low P conditions (Figure 7), which is consistent with our other experimental results (Figure 4). These results reveal NPA treatment affected root cell expansion and actin filament skewness in *PGX2^AT^* seedlings.

## Discussion

Several Arabidopsis PG genes from the large polygalacturonase gene family have been recently reported to function in hypocotyl elongation, leaf and flower morphogenesis, pollen and pollen tube development, and stem growth (Ogawa et al., 2009; Xiao et al., 2014, 2017; Rui et al., 2017; Hocq et al., 2020; Yang et al., 2021). Importantly, we previously reported that the polygalacturonase gene, *PGX2*, affects primary root length (Xiao et al., 2017). However, there are no reports on the functions of PG genes in root hair formation. In this study, we found that *PGX2^AT^* seedlings had a bubble-like root hair phenotype in response to phosphate deficiency (Figure 1). Under low phosphate conditions, plants can remodel their root systems, including by regulating root hair development, to acquire more nutrients (Bates and Lynch, 1995; Lin et al., 2020). The reorganization of root system architecture is largely dependent on the remodeling of cell walls (Williamson et al., 2001; Ogden et al., 2018). Among different cell wall components, pectin is highly hydrated and flexible, which assists its role in controlling cell expansion and/or elongation during root growth (Levesque-Tremblay et al., 2015; Geng et al., 2017; Schoenaers et al., 2018). Here, we found that P-deficient stress triggers the formation of bubble-like root hairs in *PGX2^AT^* seedlings, which was confirmed by the application of pectinase *in vitro* (Figure 1). These data indicate that P deficiency enhances the PGX2-dependent cell wall loosening in root hairs. *PGX2^AT^* plants exhibited different stress responses, including decreased callose and anthocyanin deposition, and increased superoxide accumulation under P-deficient conditions (Figure 1). Together, these results suggest that PGX2 plays an important role in root hair development and that pectin degradation mediated by PGX2 is involved in multiple physiological processes.

The polygalacturonase activity of PGX2 allows it to cleave pectin molecules *in vivo* and *in vitro* (Xiao et al., 2017). Here, we found that *PGX2^AT^* seedlings had lower pectin content but higher pectin methylesterification levels, supporting the role of PGX2 in pectin degradation and modification. However, this action of PGX2 on pectin was weakened under P-deficient conditions (Figures 2A,B). Interestingly, JIM5 fluorescence intensity was higher in *PGX2^AT^* root hairs under both normal and P-deficient conditions (Figures 2C,E), but no change was observed in the JIM7 labeling pattern (Figure 2F), which indicated more demethylesterified HG in the *PGX2^AT^* than Col seedlings. The methylesterification status in root hairs was monitored by immunolabeling with specific antibodies, while the methylesterification level was measured by biochemical method from isolated cell wall materials of whole seedlings. The different pectin methylesterification degree from biochemical measurement and immunolabeling may be attributable to differential accessibility in different tissue walls in *PGX2^AT^* plants. In addition, the degree of methylesterification may vary in different parts of the plant.

Auxin acts as an organizing node that coordinates environmental cues to regulate root hair development, and PIN proteins are a key class of factors controlling the polar growth of root hairs (Blilou et al., 2005; Michniewicz et al., 2007; Lee and Cho, 2013). In our study, the expression levels of genes associated with auxin signaling pathways, such as *RSL2*, *ARF7*, *ARF19*, and *PIN1*, were upregulated in *PGX2^AT^* seedlings, and their expression was further enhanced in response to low P (Figure 3A). Additionally, the expression of *PGX2* was significantly decreased in auxin signaling mutants (Figure 3B). Our results also showed that *PGX2^AT^* seedlings had more low-methylesterified HG in root hairs (Figures 2C,E) and that low P conditions promoted the expression of genes associated with auxin signaling in *PGX2^AT^* seedlings (Figure 3A). Moreover, the exogenous application of auxin NAA and auxin inhibitor NPA, which interferes with auxin signaling, limited the formation of bubble-like root hairs in *PGX2^AT^* seedlings under low P conditions (Figure 4). These results indicate a possible functional interaction between auxin and pectin (Braybrook and Peaucelle, 2013), and further exploration of the molecular mechanisms by which auxin acts on pectin to regulate wall properties will deepen our understanding of this relationship.

Polarized PIN2 localization in the plasma membrane and its trafficking has been shown to respond to low P conditions (Kumar et al., 2015). The polar distribution of PM-localized PIN2 proteins in *PGX2^AT^* root cells was obviously different from that in Col controls; PIN2 proteins were distributed uniformly around cells, unlike in Col controls (Figure 5). BFA treatment interrupted PIN2 trafficking and more BFA bodies accumulated inside the cells of *PGX2^AT^* roots under both normal and low P conditions. However, a few BFA bodies remained in the *PGX2^AT^* cells after washing, which was especially obvious under low P conditions (Figure 2). These data indicate that PGX2-mediated cell wall remodeling affects polar localization and trafficking of PIN2 proteins in root cells, and these processes respond to P deficiency. In addition, given that the alteration of localization and trafficking of PIN2 proteins in *PGX2^AT^* root cells in P-deficient conditions, PIN2 endocytosis might be influenced by PGX2 in response to P deficiency. This may be explained that loosening of the cell wall causes changes in the wall-PM connections that help regulate membrane recycling.

To better adapt to low P soil environments, plant root systems undergo structural and wall remodeling (Lopez-Bucio et al., 2002), and actin filament bundling and dynamics in cells can respond to these alterations (Kumar et al., 2015). Here, we found that *PGX2^AT^* root hair cells had disorganized but stable actin filaments after treatment with LatA under normal MS conditions. Actin filaments in *PGX2^AT^* root hair cells were more disorganized under low P conditions, possibly contributing to the deformation of root cells. Interestingly, actin filaments in both Col and *PGX2^AT^* root hair cells were more resistant to LatA treatment under low P conditions (Figures 6A–F). These data indicate that P deficiency can increase the stability of actin filaments in *PGX2^AT^* seedlings, likely as part of a compensatory effect. Through further assessment of actin filament arrangement in the root hairs, *PGX2^AT^* root hair cells had lower skewness of actin filaments than Col (Figure 6G), indicating that actin filaments were less bundled in *PGX2^AT^* root hair cells. Additionally, we also observed actin filament arrangement after NPA treatment and found that the lower skewness of actin filaments in *PGX2^AT^* was recovered (Figure 7), suggesting that NPA application increases actin filament bundling in *PGX2^AT^* root hair cells.

## Conclusion

In this study, we attempt to determine the key role of cell wall remodeling mediated by pectin degradation in plant root hair development, especially in response to low phosphate environments. Our results found that the activation tagging line *PGX2^AT^* had altered pectin content and pectin methylesterification level. In low phosphate conditions, *PGX2^AT^* seedlings displayed a bubble-like root hair phenotype, which was consistent with the pectinase treatment *in vitro*. Hormone auxin and cytoskeleton actin have been shown to play critical roles in root hair development. However, how cell wall remodeling cooperating with auxin signaling and actin in root hair development remains elusive. Our results found that the application of NAA or NPA inhibited the formation of bubble-like root hairs, and the polar localization and trafficking of the PIN2 protein was also affected in *PGX2^AT^* root cells. In addition, actin filaments were less bundled and more stable in *PGX2^AT^* root hair cells, and the decreased skewness of actin filaments in *PGX2^AT^* root hairs was recovered with NPA treatment, which also inhibited the formation of bubble-like root hairs of *PGX2^AT^* seedlings under low phosphate conditions. Taken together, our study demonstrates the molecular mechanism of how cell wall remodeling by pectin degradation, together with auxin signaling and cytoskeleton actin, cooperatively regulates root hair development in response to phosphate starvation. This provides a new view of the strategy for plants to adapt to low-nutrient environments.

## Data Availability Statement

The raw data supporting the conclusions of this article will be made available by the authors, without undue reservation.

## Author Contributions

CX designed the research. QZ, AD, MX, QL, XL, SY, XG, and SH performed experiments. QZ, AD, MX, JD, and CX analyzed the data and wrote the article. All authors contributed to the article and approved the submitted version.

## Funding

This work was supported by Joint Science and Technology Support Program of Sichuan University and Panzhihua City (2019CDPZH-19) to JD, the Fundamental Research Funds for the Central Universities (SCU2021D006), the Institutional Research Fund from Sichuan University (2020SCUNL302), and the National Natural Science Foundation of China (32170357) to CX.

## Conflict of Interest

The authors declare that the research was conducted in the absence of any commercial or financial relationships that could be construed as a potential conflict of interest.

## Publisher’s Note

All claims expressed in this article are solely those of the authors and do not necessarily represent those of their affiliated organizations, or those of the publisher, the editors and the reviewers. Any product that may be evaluated in this article, or claim that may be made by its manufacturer, is not guaranteed or endorsed by the publisher.
