# Supplementary material for: The Root Hair Development of Pectin Polygalacturonase PGX2 Activation Tagging Line in Response to Phosphate Deficiency
Source: Front Plant Sci. 2022 May 2;13:862171. doi: 10.3389/fpls.2022.862171 (PMC9108675; doi:10.3389/fpls.2022.862171)
Supplement: Supplementary file 1 [file Table_1.DOCX]

Supplementary Table 1. Primers used in this study.

| Gene | Primer name | Sequences (5’→3‘) | Note |
| --- | --- | --- | --- |
| AT3G26610 | PGX1-qF | GGGATTACAGACCCACCAGC | qPCR |
|  | PGX1-qR | TGGAAGATGGCACTAGGGGA | qPCR |
| AT1G48100 | PGX3-qF | AAGTCCACCGATTCATTTCG | qPCR |
|  | PGX3-qR | TCCGGCAATAACTCAACCTC | qPCR |
| AT1G78400 | PGX2-qF | TGCCGCCAAACCCATAAT | qPCR |
|  | PGX2-qR | CGAACTGGAACCCCATGTTC | qPCR |
| AT5G13080 | WRKY75-qF | GCTGCATCAAGGCGAAGAAG | qPCR |
|  | WRKY75-qR | CTTTTTCGACGACTCCACGC | qPCR |
| AT5G23630 | PDR2-qF | AATGGCATCCCCCTTCACAG | qPCR |
|  | PDR2-qR | TGTGGCGAGACAGTTCAGAC | qPCR |
| AT1G23010 | LPR1-qF | ACGGTACATCACGGAGCAAG | qPCR |
|  | LPR1-qR | GGGTCCCAAGGAAGGATGTG | qPCR |
| AT1G66470 | RHD6-qF | GAGCACTCAACCGTCGAAGA | qPCR |
|  | RHD6-qR | TCGATTCTTGGCTGCTAGGC | qPCR |
| AT1G20090 | ROP2-qF | TGTTTGTTTCCGATCTTGCGG | qPCR |
|  | ROP2-qR | CATCTCCGACGGTCACACAC | qPCR |
| AT4G33880 | RSL2-qF | ACCAGCTTCTCGCCTCTTATG | qPCR |
|  | RSL2-qR | ACACGAGGCTAAGGTGGTTG | qPCR |
| AT1G64440 | RHD1-qF | GGTTGGAAGGAGACCAGGTG | qPCR |
|  | RHD1-qR | TTGCCCAGTTCCACTGATCC | qPCR |
| AT5G20350 | TIP1-qF | TCTGTCGCCGTCTTAACAGTC | qPCR |
|  | TIP1-qR | GGGAAGCAAAGTACGAAAGAGC | qPCR |
| AT2G38120 | AUX1-qF | CCCATCTCTCGCTCACATGC | qPCR |
|  | AUX1-qR | CTTCTCCGCCGCATTCTGAC | qPCR |
| AT1G70940 | PIN3-qF | GTAGCCTCGAGTGGAGCATC | qPCR |
|  | PIN3-qR | GTTGGGAAGTGTGGAGAGGG | qPCR |
| AT1G73590 | PIN1-qF | CTCGCTTACGGCTCTGTCAA | qPCR |
|  | PIN1-qR | CGGTTTATGCCGGAGCATTG | qPCR |
| AT5G57090 | PIN2-qF | TTCTTTGGCAGGCGTTTAGC | qPCR |
|  | PIN2-qR | ACCAACGTGTTAGGCAGTGT | qPCR |
| AT5G61350 | ERU-qF | CGTGACCATCTCTACGGCTC | qPCR |
|  | ERU-qR | ACCCGTGTGGAGATAGTGGA | qPCR |
| AT1G14130 | DAO1-qF | GTTGCCAAACACGCTTGCTA | qPCR |
|  | DAO1-qR | GCACAATCTTCCGTTGCTCC | qPCR |
| AT5G20730 | ARF7-qF | TGCAACAGCTGCCTACTCTC | qPCR |
|  | ARF7-qR | CCAGCATTTGAGGTGGTTGC | qPCR |
| AT1G19220 | ARF19-qF | GAGAGCCGGATCAGTCAAGG | qPCR |
|  | ARF19-qR | CAGGACAGTACGTCGTTCCC | qPCR |
| AT5G54380 | THE1-qF | AAGGCGGTAATGGACATCCG | qPCR |
|  | THE1-qR | AGCTGTGGCACTCTTGTGAG | qPCR |
| AT1G58200 | MSL3-qF | TGGCACAGTAGAGCAAGTCG | qPCR |
|  | MSL3-qR | CTTCCCGGTCATCACCTCTG | qPCR |
| AT3G51550 | FER-qF | GACCAGGGCATTGAGAGACC | qPCR |
|  | FER-qR | TCTTCTGCGCTTTCCTGGAG | qPCR |
| AT1G18070 | EF1α-qF | TGGTGACGCTGGTATGGTTA | qPCR |
|  | EF1α-qR | TCCTTCTTGTCCACGCTCTT | qPCR |
| At3G18780 | ACT2-qF | CTTGCACCAAGCAGCATGAA | qPCR |
|  | ACT2-qR | CCGATCCAGACACTGTACTTCCTT | qPCR |
