# Supplementary material for: The Root Hair Development of Pectin Polygalacturonase PGX2 Activation Tagging Line in Response to Phosphate Deficiency
Source: Front Plant Sci. 2022 May 2;13:862171. doi: 10.3389/fpls.2022.862171 (PMC9108675; doi:10.3389/fpls.2022.862171)
Supplement: Supplementary file 2 [file Table_2.DOCX]

Supplementary Table 2. Genes for expression level detection in Col and *PGX2^AT^* seedlings.

| **Name** | **Gene** | **Description** | **Reference** |
| --- | --- | --- | --- |
| **phosphate deficiency response** | | | |
| *WRKY 75* | At5g13080 | a transcription factor of phosphate acquisition and root development | (Devaiah et al., 2007; Rishmawi et al., 2014) |
| *PDR2* | At5g23630 | a single P5-type ATPase required for SCN and RAM maintenance in Pi-deprived roots | (Muller et al., 2015) |
| *LPR1* | *At1g23010* | a multicopper oxidase required for SCN and RAM maintenance in Pi-deprived roots | (Muller et al., 2015) |
| **root hair development** | | | |
| *RHD6* | At1g66470 | required for early stages of trichoblast development | (Moro et al., 2017) |
| *ROP2* | At1g20090 | activated by auxin, a positive regulator of root hair initiation and tip growth | (Kang et al., 2017) |
| *RSL2* | At4g33880 | crucial for hair morphogenesis, responsive to P deficiency | (Bhosale et al., 2018) |
| *RHD1* | At1g64440 | necessary for proper initiation of root hairs | (Schiefelbein and Somerville, 1990) |
| *TIP1* | At5g20350 | play a key role in growth polarity, mutants exhibit root hairs with multiple initiations and with branches. | (Zhang et al., 2015; Chai et al., 2016) |
| **auxin transport and signaling** | | | |
| *AUX1* | At2g38120 | auxin transport | (Qin and Huang, 2018) |
| *PIN3* | At1g70940 | Auxin efflux carrier family protein | (Sasayama et al., 2013) |
| *PIN1* | At1g73590 | Auxin efflux carrier family protein | (Sasayama et al., 2013) |
| *PIN2* | At5g57090 | Auxin efflux carrier family protein | (Rigo et al., 2013) |
| *ERU* | At5g61350 | regulate cell wall composition in root hairs and modulates pectin dynamics through negative controlling pectin methylesterase (PME) activity | (Schoenaers et al., 2018) |
| *DAO1* | At1g14130 | up-regulated by P deficiency | (Bhosale et al., 2018) |
| *ARF7* | At5g20730 | a transcription factor of root hair growth | (Schoenaers et al., 2018) |
| *ARF19* | At1g19220 | a transcription factor of root hair growth | (Schoenaers et al., 2018) |
| **cell wall integrity signaling** | | | |
| *THE1* | At5g54380 | a key signaling element mediating CWD | (Engelsdorf et al., 2018) |
| *MSL3* | At1g58200 | mechanosensitive, activation of CWD-induced responses | (Engelsdorf et al., 2018) |
| *FER* | At3g51550 | CWD perception, required during root hair development | (Engelsdorf et al., 2019) |

**REFERENCES**

Bhosale, R., Giri, J., Pandey, B.K., Giehl, R.F.H., Hartmann, A., Traini, R., et al. (2018). A mechanistic framework for auxin dependent Arabidopsis root hair elongation to low external phosphate. *Nat Commun* 9, 1-9. doi:10.1038/S41467-018-03851-3

Chai, S., Ge, F.R., Feng, Q.N., Li, S., and Zhang, Y. (2016). PLURIPETALA mediates ROP2 localization and stability in parallel to SCN1 but synergistically with TIP1 in root hairs. *Plant J* 86, 413-425. doi:10.1111/tpj.13179

Devaiah, B.N., Karthikeyan, A.S., and Raghothama, K.G. (2007). WRKY75 transcription factor is a modulator of phosphate acquisition and root development in arabidopsis. *Plant Physiol* 143, 1789-1801. doi:10.1104/pp.106.093971

Engelsdorf, T., Gigli-Bisceglia, N., Veerabagu, M., McKenna, J.F., Vaahtera, L., Augstein, F., et al. (2018). The plant cell wall integrity maintenance and immune signaling systems cooperate to control stress responses in Arabidopsis thaliana. *Science Signaling* 11, 1-14. doi:10.1126/scisignal.aao3070

Engelsdorf, T., Kjaer, L., Gigli-Bisceglia, N., Vaahtera, L., Bauer, S., Miedes, E., et al. (2019). Functional characterization of genes mediating cell wall metabolism and responses to plant cell wall integrity impairment. *BMC Plant Biol* 19, 1-15. doi:10.1186/s12870-019-1934-4

Kang, E., Zheng, M., Zhang, Y., Yuan, M., Yalovsky, S., Zhu, L., et al. (2017). The microtubule-associated protein MAP18 affects ROP2 GTPase activity during root hair growth. *Plant Physiol* 174, 202-222. doi:10.1104/pp.16.01243

Moro, C.F., Gaspar, M., da Silva, F.R., Pattathil, S., Hahn, M.G., Salgado, I., et al. (2017). S-nitrosoglutathione promotes cell wall remodelling, alters the transcriptional profile and induces root hair formation in the hairless root hair defective 6 (rhd6) mutant of Arabidopsis thaliana. *New Phytol* 213, 1771-1786. doi:10.1111/nph.14309

Muller, J., Toev, T., Heisters, M., Teller, J., Moore, K.L., Hause, G., et al. (2015). Iron-dependent callose deposition adjusts root meristem maintenance to phosphate availability. *Developmental Cell* 33, 216-230. doi:10.1016/j.devcel.2015.02.007

Qin, H., and Huang, R. (2018). Auxin controlled by ethylene steers root development. *International Journal of Molecular Sciences* 19, 1-13. doi:10.3390/ijms19113656

Rigo, G., Ayaydin, F., Tietz, O., Zsigmond, L., Kovacs, H., Pay, A., et al. (2013). Inactivation of plasma membrane-localized CDPK-RELATED KINASE5 decelerates PIN2 exocytosis and root gravitropic response in Arabidopsis. *Plant Cell* 25, 1592-1608. doi:10.1105/tpc.113.110452

Rishmawi, L., Pesch, M., Juengst, C., Schauss, A.C., Schrader, A., and Hulskamp, M. (2014). Non-cell-autonomous regulation of root hair patterning genes by WRKY75 in Arabidopsis. *Plant Physiol* 165, 186-195. doi:10.1104/pp.113.233775

Sasayama, D., Ganguly, A., Park, M., and Cho, H.T. (2013). The M3 phosphorylation motif has been functionally conserved for intracellular trafficking of long-looped PIN-FORMEDs in the Arabidopsis root hair cell. *BMC Plant Biol* 13, 1-11. doi:10.1186/1471-2229-13-189

Schiefelbein, J.W., and Somerville, C. (1990). Genetic control of root hair development in Arabidopsis thaliana. *Plant Cell* 2, 235-243. doi:10.1105/tpc.2.3.235

Schoenaers, S., Balcerowicz, D., Breen, G., Hill, K., Zdanio, M., Mouille, G., et al. (2018). The auxin-regulated CrRLK1L kinase ERULUS controls cell wall composition during root hair tip growth. *Curr Biol* 28, 722-732. doi:10.1016/j.cub.2018.01.050

Zhang, Y.L., Li, E., Feng, Q.N., Zhao, X.Y., Ge, F.R., Zhang, Y., et al. (2015). Protein palmitoylation is critical for the polar growth of root hairs in Arabidopsis. *BMC Plant Biol* 15, 1-12. doi:10.1186/s12870-015-0441-5
