# Supplementary material for: The Root Hair Development of Pectin Polygalacturonase PGX2 Activation Tagging Line in Response to Phosphate Deficiency
Source: Front Plant Sci. 2022 May 2;13:862171. doi: 10.3389/fpls.2022.862171 (PMC9108675; doi:10.3389/fpls.2022.862171)
Supplement: Supplementary file 3 [file Data_Sheet_1.PDF]

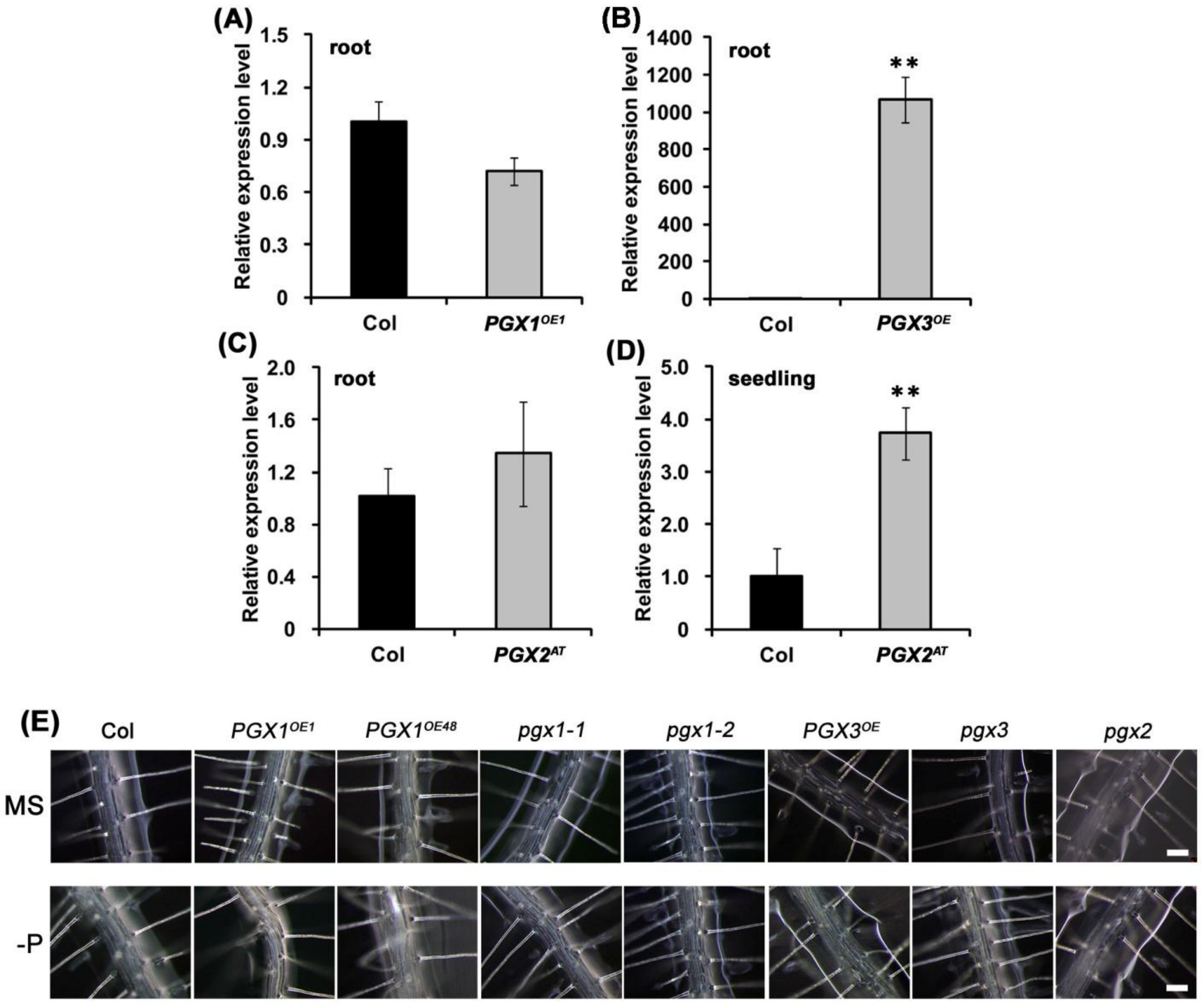

Supplementary Figure 1. Gene expression detection and root hairs of Col and PG-related mutants grown under phosphate deficient conditions. **(A-D)** Gene expression detection by qPCR from the roots and seedlings of seven-day-old light-grown Col, *PGX1*^{OE1}, *PGX3*^{OE}, *PGX2*^{AT} plants (n = 3 technical replicates, *t*-test, **P < 0.001). **(E)** Primary roots with root hair region of Col, *PGX1*^{OE1}, *PGX1*^{OE48}, *pgx1-1*, *pgx1-2*, *PGX3*^{OE}, *pgx3* and *pgx2* seedlings grown in normal (MS, 480 μM) and phosphate deficient (-P, 10 μM) conditions. Images focusing on root hair regions were taken after 3-day-old seedlings were transferred onto normal MS or phosphate deficient medium plates for another four days. Bars = 100 μm.

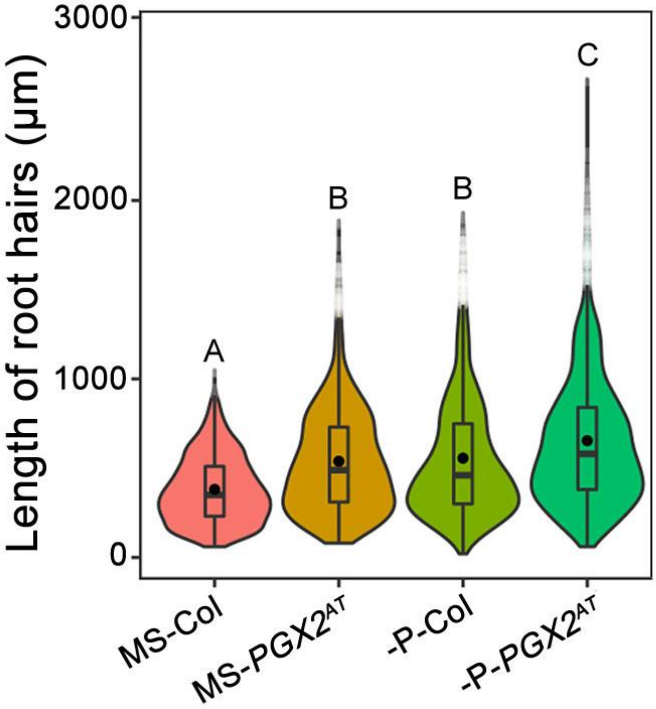

Supplementary Figure 2. *PGX2^{AT}* seedlings have longer root hairs under normal and phosphate deficient conditions. Violin plot of root hair length of seven-day-old Col and *PGX2^{AT}* seedlings under normal and low P conditions (n ≥ 800 root hairs from at least 10 seedlings). Uppercase letters indicate significantly different groups as determined by one-way ANOVA with post-hoc Duncan's test (P < 0.01).

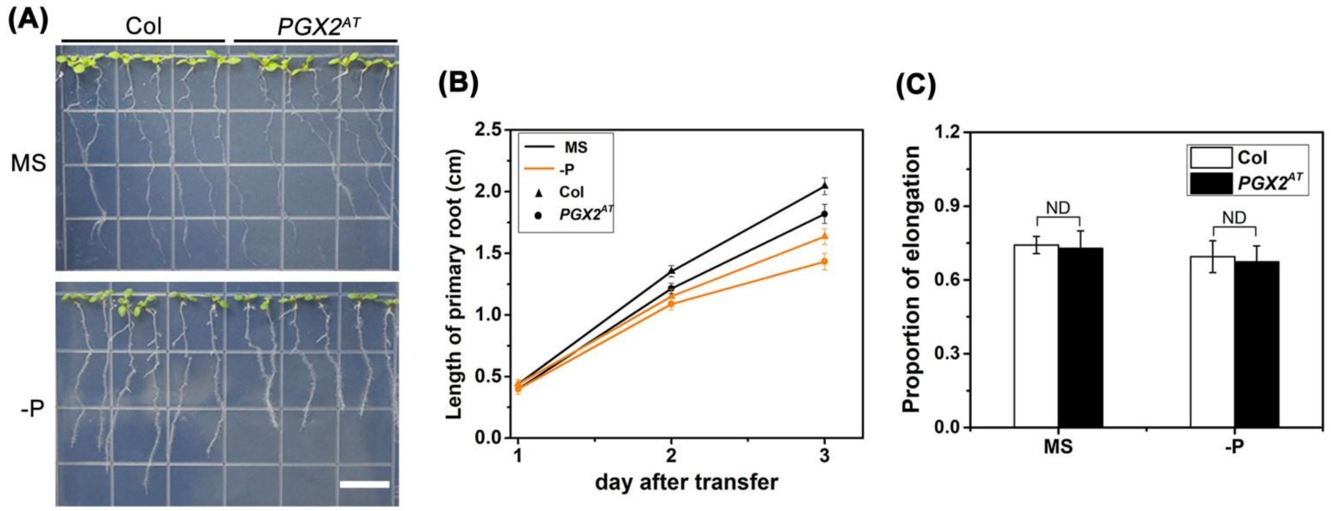

Supplementary Figure 3. Primary root length of Col and *PGX2*^{AT} seedlings. **(A)** Col and *PGX2*^{AT} seedlings grown for three days in normal conditions were transferred onto normal MS or phosphate deficient (-P) MS medium plates for another four days. Bar = 1 cm. **(B)** Primary root lengths of Col and *PGX2*^{AT} seedlings after seedlings were transferred to normal MS and -P conditions. **(C)** Proportion of root elongation to total length of Col and *PGX2*^{AT} seedlings (n = 35 seedlings per each genotype). Error bars represent SD. ND, no statistical difference.

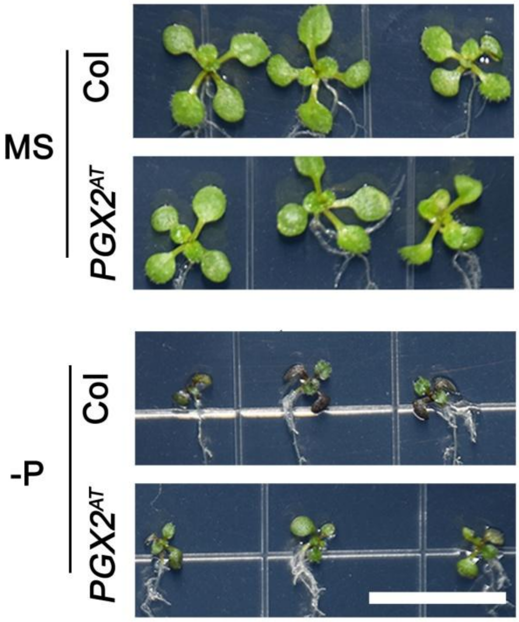

Supplementary Figure 4. Anthocyanin accumulation in leaves of Col and *PGX2*^{AT} seedlings under low phosphate conditions. *PGX2*^{AT} seedlings display less anthocyanin accumulation compared to Col controls against low phosphate treatment. Images were taken in the 10th day after seedling transfer. Bar = 1 cm.

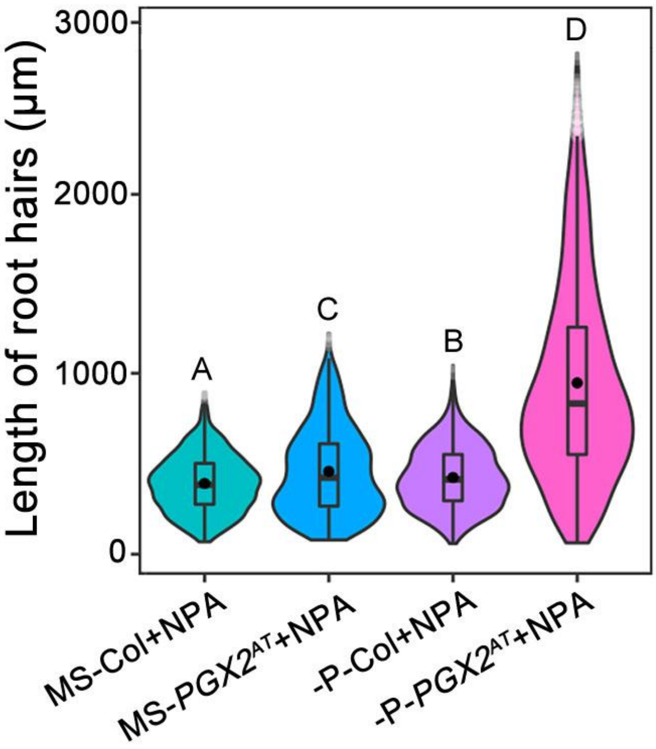

Supplementary Figure 5. NPA application promotes root hair elongation of *PGX2*^{AT} seedlings in phosphate deficient conditions. Violin plot of root hair length of seven-day-old Col and *PGX2*^{AT} seedlings with NPA treatment under normal and low P conditions (n ≥ 800 root hairs from at least 10 seedlings). Uppercase letters indicate significantly different groups as determined by one-way ANOVA with post-hoc Duncan’s test ($P < 0.01$).

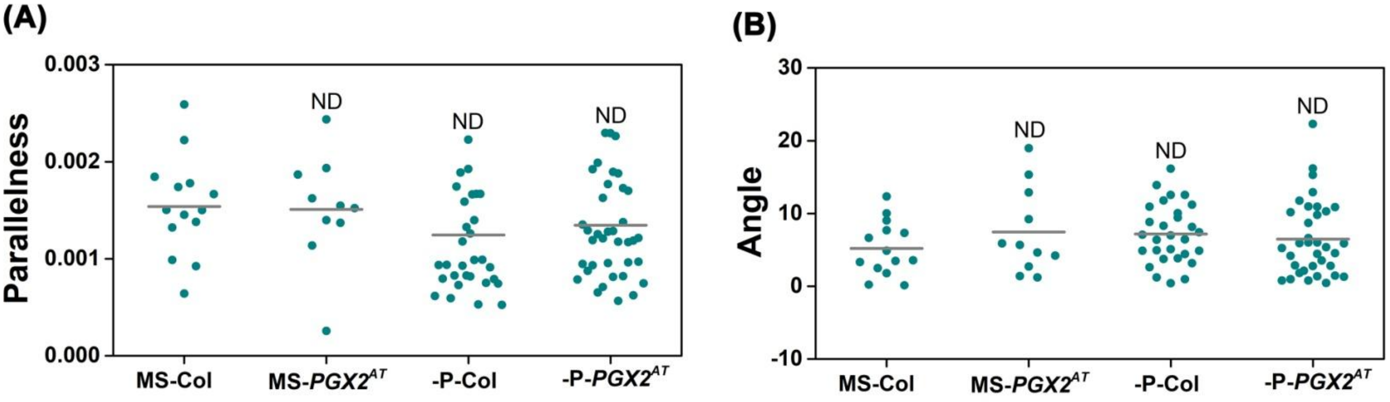

Supplementary Figure 6. Actin filament orientation in *Lifeact-GFP* Col and *Lifeact-GFP PGX2^{AT}* transgenic seedlings under normal MS and phosphate deficient conditions. **(A, B)** Parallelness and angle of actin filaments relative to cell elongation axis (n ≥ 11 cells). ND, no statistical difference.
